# Effectiveness of Grounded Sleeping on Recovery After Intensive Eccentric Muscle Loading

**DOI:** 10.3389/fphys.2019.00035

**Published:** 2019-01-28

**Authors:** Erich Müller, Patrick Pröller, Fatima Ferreira-Briza, Lorenz Aglas, Thomas Stöggl

**Affiliations:** ^1^Department of Sport and Exercise Science, University of Salzburg, Salzburg, Austria; ^2^Olympic Training Center Salzburg-Rif, Hallein, Austria; ^3^Department of Biosciences, University of Salzburg, Salzburg, Austria

**Keywords:** creatine kinase (CK), downhill running, inflammation, muscle soreness, muscle strength

## Abstract

**Purpose:** We set out to investigate the effectiveness of grounded sleeping on the time course of recovery with respect to muscle soreness and athletic performance after intensive eccentric muscle loading.

**Methods:** Twenty-two healthy participants were recruited for this study and randomly assigned to an experimental group (GRD, grounded sleeping, *n* = 12) or control group (UGD, sham-grounded sleeping, *n* = 10) to evaluate the effects of 10 days recovery with GRD vs. UGD following a single intensive downhill treadmill intervention in a triple-blinded (participant, tester, and data analyst) manner. To operationalize recovery a test battery was performed at baseline and on days 1, 2, 3, 5, 7, and 10 post-intervention: (1) perception of muscle soreness (VAS), (2) creatine kinase blood levels (CK), (3) maximum voluntary isometric contraction (MVIC) for both legs, (4) counter movement jump (CMJ) and drop jump (DJ) performance. Furthermore, in four participants blood was sampled for detailed analysis of complete blood counts and serum-derived inflammation markers.

**Results:** The downhill treadmill running intervention led to distinct changes in all measured parameters related to fatigue. These changes were detectable already 5-min post intervention and were not fully recovered 10 days post intervention. GRD led to less pronounced decrease in performance (CMJ, MVIC) and less increase with respect to CK compared with UGD (all *P* < 0.05). Detailed blood samples demonstrated that grounded sleeping modulates the recovery process by (a) keeping a constant hemoconcentration, as represented by the number of erythrocytes, and the hemoglobin/hematocrit values; and (b) by the reduction of muscle damage-associated inflammation markers such as, IP-10, MIP-1α, and sP-Selectin.

**Conclusion:** The downhill running protocol is a feasible methodology to produce long term muscle soreness and muscular fatigue. GRD was shown to result in faster recovery and/or less pronounced markers of muscle damage and inflammation. GRD might be seen as a simple methodology to enhance acute and long-term recovery after intensive eccentric exercises.

## Introduction

Recovery following intense training bouts is crucial for both professional and recreational athletes. It is well understood that exercise-induced muscle damage (EIMD) usually follows novel, unaccustomed repetitive movements and/or strenuous eccentric contractions (Proske and Morgan, 2001; Proske et al., 2004; Mackey et al., 2008). This kind of external load induces muscle damage that is associated with delayed onset muscle soreness (DOMS) (Hough, 1902).

Exercise-induced muscle damage is often associated with temporary substantial drops in performance, local muscle soreness and enhanced risk for musculoskeletal injuries. In professional sports, athletes have to recover within a few days to regain their performance levels. In spite of both the frequency and monetary consequences of DOMS in elite athletes, the de facto underlying mechanisms and their impacts on performance and treatment strategies remain vague (Cheung et al., 2003). A number of conventional therapeutic strategies are applied to alleviate clinical symptoms of DOMS. Nonetheless, Cheung et al. (2003) and Seidel et al. (2012) concluded that, in fact, no treatment strategy consistently supported or enhanced muscle recovery, which indicates that there is actually a lack of compelling evidence-based and practical strategies to help prevent and/or alleviate DOMS.

Grounding, earthing, or grounded sleeping, is a process in which the athlete becomes grounded via an electrically conducted device. The person is grounded in an indirect way that corresponds to being barefoot with direct, continuous contact with the earth. Nowadays, it is almost impossible to be earthed, due to urbanization, insulating footwear or bituminization. There are various grounding systems available that permit contact with the surface of the Earth. This indirect way of earthing or grounding is based on trivial conductive systems like sheets, mats, wrist or ankle bands, adhesive patches that can be used during sleeping or working, or inside footwear. These devices get coupled to the Earth by a typical cord slotted into a grounded wall outlet (Oschman et al., 2015). Referring to Chevalier et al. (2006); Oschman (2007), and Oschman et al. (2015) the main hypothesis about earthing is based on the connection to the surface of the Earth, which is satiated with free electrons. This indirect or direct contact with the Earth enables “mobile” electrons to migrate into the body. Oschman (2007) suggests that these free electrons act as antioxidants in the organism and could neutralize reactive oxygen species (ROS). ROS are byproducts of mitochondrial metabolism of oxygen and delivered by the oxidative burst as part of the inflammation response. Harman (1956, 2009) postulated that these reactive chemical species are linked to the aging process, originally known as free-radical theory of aging, and in the 1970s was extended to the mitochondrial theory of aging. Over the past several years, an emerging body of evidence is starting to indicate that ROS are associated with tumorigenesis, cancer and chronic inflammatory systemic diseases (Reddy and Clark, 2004; Waris and Ahsan, 2006; Gupta et al., 2012). Consequently, Oschman (2007) suggests that the earthing based mobile electrons could also prevent or diminish inflammation.

Actually, evidence-based research regarding the effectiveness of grounding is lacking. The narrative review of Chevalier et al. (2012) included grounding studies that indicated improvements in sleep (Ghaly and Teplitz, 2004), indices of DOMS (Brown et al., 2010; Brown et al., 2015), autonomic tone (Sinatra, 2011) and reduction in blood viscosity (Chevalier et al., 2013; Brown and Chevalier, 2015). Due to the potentially reduced blood viscosity, enhanced blood flow velocity, improved sleep quality and decreased muscle damage, it is suggested that grounding could be implemented as a viable, effective recovery tool after strenuous exercise. However, currently, the evidence of treatment strategies to accelerate recovery after EIMD is still inconsistent (Connolly et al., 2003).

Therefore, the aim of this study was to investigate the effectiveness of grounded sleeping on the time course of DOMS and athletic performance after intensive downhill running. We hypothesized that grounded sleeping would alleviate exercise induced muscle and accelerate the recovery of athletic performance after strenuous downhill running.

## Materials and Methods

### Participants

Twenty-two healthy sport science and physiotherapy students (mean ± SD; *n* = 22, 10 women, 12 men, age: 23.8 ± 3.2 years, weight: 67.2 ± 7.6 kg, height: 174.2 ± 6.3 cm) were recruited for this study. All Participants were free of musculoskeletal disorders, cardiovascular diseases, dietary supplements or prescription medicines that could potentially affect muscle recovery. All participants received detailed oral and written information about the procedures and the possible risks, and gave their written consent to participate. The study received approval from the local Ethics Committee of the University of Salzburg (Kapitelgasse 4, 5020 Salzburg, GZ 13/2017) and was conducted in accordance with the Declaration of Helsinki. Participants were given grounding equipment for compensation. Participants were randomly assigned to the experimental group (GRD, grounded sleeping, *n* = 12) or the control group (UGD, sham-grounded sleeping, *n* = 10). No difference in any baseline values between groups were detected (Table 1).

**Table 1:** Physical characteristics of the participants in the grounded sleeping group (GRD) versus the sham-grounded group (UGD) (mean ± SD).

		Body			DJ-Coefficient
Group	Height (m)	mass (kg)	Age (years)	CMJ-Height (cm)	(height/time)	MVIC-R (*N*)	MVIC-L (*N*)	CK (U/L)	VAS (6–20)
**GRD**	177 ± 8	71 ± 10	23.9 ± 4.1	29.2 ± 6.0	140 ± 43	2029 ± 749	1986 ± 780	261 ± 168	4.6 ± 4.3
**UGD**	174 ± 7	67 ± 9	23.7 ± 2.3	27.4 ± 7.4	115 ± 28	1713 ± 507	1596 ± 539	154 ± 99	3.7 ± 4.0

*SJ, squat jump; DJ, drop jump; MVIC-R, MVIC-L, maximum isometric voluntary contraction right-left leg; CK, creatine kinase; VAS, visual analog scale.*

### Study Design

**Figure 1:**
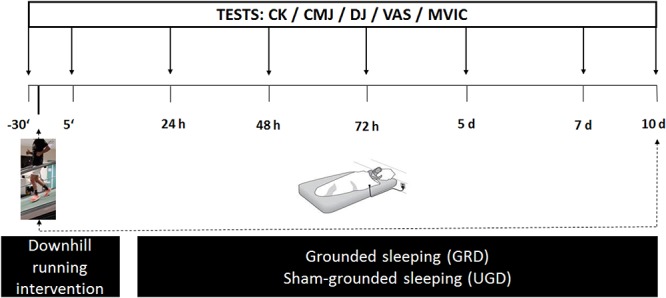
Schematic illustration of the procedure with a timeline: pretest (–30 min prior to intervention), intervention (downhill running), posttests (+5 min, 24 h, 48 h, 72 h, 5 days, 7 days, and 10 days post intervention) consisting determination of blood creatine kinase content (CK), visual analog scale (VAS) related to muscle soreness, drop jump (DJ) performance, counter movement jump performance (CMJ) and a maximum voluntary isometric contraction (MVIC) of both the left and right leg.

A triple-blinded (participant, tester, and data analyst) randomized controlled design was used to evaluate the effects of 10 days recovery including grounded sleeping (GRD) or sham-grounded sleeping (UGD) after a single intensive downhill treadmill running intervention designed to induce DOMS. To operationalize recovery a test battery was performed before the intervention (baseline) and on days 1, 2, 3, 5, 7, and 10 post intervention. This test battery included: perception of muscle soreness via a visual analog scale (VAS), creatine kinase blood levels (CK), maximum voluntary isometric contraction (MVIC) for both legs, counter movement jump (CMJ) and drop jump (DJ) performance (see Figure 1). Furthermore, as a pilot study in four participants (GRD, *n* = 2; and UGD, *n* = 2), blood was sampled for detailed analysis of complete blood counts and serum-derived inflammation markers.

### Instruments and Procedures

#### Downhill Running

All participants underwent a 20-min exercise bout of intensive downhill running (-25% slope, 12 km ⋅ h^-1^) on a motorized treadmill (Saturn 300/100 rs, h/p/cosmos sports & medical GmbH, Germany). For determination of the physiological and psychological loading of the intervention, heart rate (Suunto Ambit 3.0, Helsinki, Finland), blood lactate (Biosen S-Line EFK Diagnostik, Germany) in the 1st, 3rd, and 5th minute post intervention and rating of perceived exertion (BORG, 6–20) in the last minute of running were collected.

#### Grounded or Sham-Grounded Sleeping

During the 10-day post intervention, the GRD group slept grounded with a conductive sheet connected with a grounded wall outlet, while the UGD group slept sham-grounded. Both groups received identical sheets provided by BTZ (Badisches Therapie Zentrum, Baden-Baden, Germany) prior to the start of the study. These sheets (sized 90 cm × 200 cm) are made of 100% cotton with conductive silver fibers woven into the fabric. The sheet attaches to a grounded cord that connects at the other end to the ground of the wall socket. There is no direct connection to electricity; therefore, grounding via an indirect manner (e.g., cable) is safe and does not pose any danger to the individuals. To establish the control situation and subsequent blinding, the grounding plugs were manipulated and masked by an independent person. Neither the investigator, nor the participants were aware of this modification of the grounded system. The participants were instructed to sleep grounded at home on their sheets, ensuring as much skin contact as possible. Additionally, the participants were consistently reminded that it would be best if they slept either naked, or at most, wear no more than underwear. The participants were allowed to sleep with a pillow that was not grounded. In the event that a participant was not to sleep at home on any of the 10 days, they were advised to take the sheet with them.

A pilot study (*n* = 10) about the effects of earthing with the conductive sheets (grounded vs. sham-grounded) was performed prior to start of the main study. Participants were asked to lay down on a massage bed and relax on both the grounded139 or sham-grounded sheet for 5-min each wearing only underwear. Electrostatic charge was measured on the skin at the region of the vastus lateralis via an electrostatic voltmeter (ESVM 1000, Wolfgang Warmbier, Germany). The pilot study revealed values of -0.2 ± 0.1 V vs. -81.9 ± 25.6 V (*P* < 0.001) in the grounded vs. sham-grounded situation clearly demonstrating the effects of grounding via the conductive sheet.

#### Counter-Movement-Jump and Drop-Jump

The CMJ was executed without arm-swing and the DJ was performed from a 40 cm dropping height. Both jump modes were performed on a force plate (BP600900, AMTI, United States) to evaluate the maximum jumping height (CMJ and DJ) and the ground contact time and coefficient between jump height and ground contact time during the DJ. The best of three attempts were used for further analysis.

#### Maximum Voluntary Isometric Contraction (MVIC)

The MVIC of the left and the right leg was assessed in a self-constructed unilateral horizontal leg press with an integrated load cell (Hottinger Baldwin Messtechnik GmbH, Germany). The participants were seated with 110° knee flexion angle (180° equivalent to full knee extension) and were allowed to grab the handles. The individual set up of the knee angle was documented with a goniometer to ensure identical settings at baseline and at follow-ups. The best of three attempts was used for further analysis. The stronger leg at baseline was defined as the dominant leg. The researchers motivated verbally during the execution.

#### Creatine Kinase (CK)

The CK enzyme is one of the most common objective reported indices of DOMS that is associated with muscle damage (Eston et al., 1994; Urhausen et al., 1995; Brown et al., 2015; Saw et al., 2016). For analysis of CK a capillary blood draw of 32 μl was taken and analyzed with the Reflotron Sprint system (Roche Diagnostics GmbH, Germany).

#### Perception of the Muscle Soreness (VAS)

The VAS was used to evaluate the muscle soreness of the lower extremity. The length of the VAS was 100 mm, and the participants had to fill in the scale at the beginning of each test day. The VAS is commonly used to assess the muscle soreness and is a valid and reliable test (Bijur et al., 2001; Gallagher et al., 2001).

#### Blood Samples

In four subjects venous blood was collected at each time point. All blood draws were performed by an experienced and certified phlebotomist. The analysis of blood counts (erythrocytes, hemoglobin, hematocrit, MCV, MCH, MCHC, leucocytes, monocytes, granulocytes, platelets and lymphocytes) was performed using a Celltac MEK-6500 (EuroLAB, Hallein, Austria). Serum-derived inflammation markers including sE-selectin, GM-CSF, sICAM-1/CD54, IFNα, IFNγ, IL-1α, IL-1β, IL-4, IL-6, IL-8, IL-10, IL-12p70, IL-13, IL-17A/CTLA-8, IP-10/CXCL10, MCP-1/CCL2, MIP-1α/CCL3, MIP-1β/CCL4, sP-selectin, and TNF alpha were analyzed with a Luminex^®^ MAGPIX^®^ System (Luminex Corporation, Austin, TX, United States) using a 20-Plex Human ProcartaPlex Panel^TM^ (Thermo Fisher Scientific, Waltham, MA, United States). The C-reactive protein (CRP) value was determined from full blood using a EUROLyser CUBE-S (EUROLyser Diagnostica GmbH, Salzburg, Austria). Raw data were normalized to percentage difference based on the baseline value (day 0), equation: 100^∗^(value-baseline)/baseline.

#### Statistical Analysis

All data exhibited a Gaussian distribution verified by the Shapiro–Wilk’s test and accordingly, the values are presented as means (±SD). The effects of the downhill treadmill running intervention on the various test parameters were analyzed with paired sampled *t*-tests between baseline and 5-min post intervention values. All post intervention test data where grounded or sham-grounded overnight sleeping has already taken place (days 1–10) were compared as how they changed from the baseline levels in per cent (day 0). A 2 × 6 ANOVA with repeated measures was used to compare both groups (GRD versus UGD) over the six-time points (days 1, 2, 3, 5, 7, and 10). The blood data were analyzed using the GraphPad Prism 7 software (La Jolla, CA, United States). Statistical analysis of blood values and inflammation markers was performed using an unpaired *t*-test with Welch’s correction. Alpha level of significance was set to 0.05. In addition, the values obtained were evaluated by calculating the effect size ($\eta_{p}^{2}$) and statistical power. The Statistical Package for the Social Sciences (Version 24.0; SPSS Inc., Chicago, IL, United States) was used for statistical analysis.

## Results

In both groups the intensive downhill treadmill protocol led to high physiological (e.g., heart rate of 200 bpm) and psychological loading (RPE between 18 and 19) with slightly higher response in UGD compared with GRD with respect to peak blood lactate (5.1 versus 3.2 mmol ⋅ L^-1^, *P* = 0.047) and RPE (18.9 vs. 18.3, *P* = 0.033) (Table 2). The intervention led to distinct reductions in jump performance (CMJ jump height, DJ jump height, DJ coefficient, all *P* < 0.001), MVIC during leg extension (both legs *P* < 0.001) and increases in VAS associated with muscle soreness (*P* < 0.001). CK levels were increased in both GRD (*P* = 0.003) and UGD (*P* = 0.024).

**Table 2:** Physical and psychological exertion during the treadmill downhill running intervention in the grounded sleeping (GRD) and sham-grounded sleeping (UGD) group (mean ± SD).

	LA_peak_ (mmol ⋅ L^-1^)	HR_max_ (bpm)	HR_mean_ (bpm)	RPE (6–20)
**GRD**	3.2 ± 1.3	201 ± 12	177 ± 12	18.3 ± 0.8
**UGD**	5.1 ± 2.9^∗^	201 ± 8	177 ± 11	18.9 ± 0.7^∗^

*LA_*peak*_, peak lactate within the first 5-min post intervention; HR_*max*_, maximal heart rate; HR_*mean*_, mean heart rate; RPE, rate of perceived exertion (6–20 Borg scale); ^∗^P < 0.05, different to GRD.*

**Figure 2:**
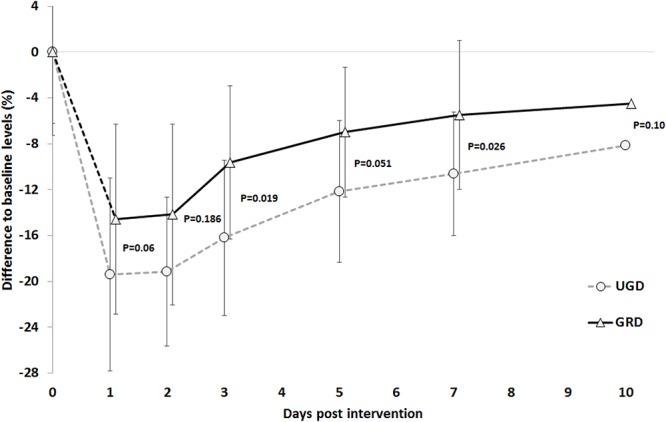
Time course of the % reductions with respect to baseline levels in the counter movement jump (CMJ) across the 10 days post intervention period. UGD, sham-grounded sleeping group; GRD, grounded sleeping group (mean ± SD).

The time course of the absolute values in measured variables across the 10-days recovery period is presented in Table 3. With respect to the CMJ jump height there was a main effect of time (*P* < 0.001, $\eta_{p}^{2}$ = 0.50, power = 1.0) and group, with a systematically lower reduction in GRD compared with UGD (-8.2 ± 5.4% vs. -14.3 ± 5.4%, *P* = 0.017, $\eta_{p}^{2}$ = 0.25, power = 0.70) but no interaction in the time course of recovery between the two groups (*P* = 0.79). Lowest CMJ performance was achieved on the 1st day post intervention (Figure 2).

**Table 3:** Measured test parameters during the 10 days recovery period after the strenuous downhill running intervention in the grounded sleeping (GRD) and sham-grounded sleeping (UGD) group (mean ± SD).

	Group	BL (-30′)	*D*_0_ (+5′)	*D*_1_ (24 h)	*D*_2_ (48 h)	*D*_3_ (72 h)	*D*_5_	*D*_7_	*D*_10_
CMJ height (cm)	UGD	27.4 ± 7.4	22.9 ± 6.7^∗∗∗^	22.2 ± 6.2^7,10^	22.1 ± 5.9^7,10^	22.9 ± 6.0^1^	24.2 ± 6.2^1,2^	24.6 ± 6.2^0,1,2^	25.1 ± 6.5^1,2^
	GRD	31.3 ± 7.5	27.9 ± 7.3^∗∗∗^	27.2 ± 7.2^7,10^	27.6 ± 7.8^7,10^	29.0 ± 8.3^7^	29.4 ± 7.5	30.1 ± 8.3^1,2,3^	30.2 ± 7.8^1,2^
DJ height (cm)	UGD	28.3 ± 5.2	22.2 ± 5.4^∗∗∗7,10^	21.2 ± 4.9^5,7,10^	20.5 ± 3.9^5,7,10^	23.0 ± 4.6	25.9 ± 4.2^1,2^	25.9 ± 4.5^0,1,2^	26.8 ± 5.1^0,1,2^
	GRD	34.2 ± 7.8	28.2 ± 7.5^∗∗∗5,7,10^	28.1 ± 7.4^5,7,10^	28.8 ± 7.9^5,7,10^	30.8 ± 9.6	32.0 ± 9.6^0,1,2^	33.4 ± 9.0^0,1,2^	32.7 ± 8.4^0,1,2^
DJ GCT (ms)	UGD	250 ± 27	272 ± 42^∗^	276 ± 50	288 ± 47^7,10^	292 ± 53^10^	274 ± 43	270 ± 42^2^	253 ± 41^2,3^
	GRD	253 ± 53	274 ± 70	262 ± 70	276 ± 61	274 ± 74	262 ± 59	259 ± 67	256 ± 59
DJ coeff (cm/s)	UGD	115 ± 28	85 ± 29^∗∗∗10^	80 ± 27^7,10^	74 ± 21^5,7,10^	82 ± 26^5,7,10^	98 ± 29^2,3^	99 ± 27^1,2,3^	110 ± 31^0,1,2,3^
	GRD	140 ± 43	110 ± 42^∗∗∗5,7,10^	116 ± 45^7^	112 ± 41^5,7,10^	122 ± 51^7^	129 ± 50^0,2^	137 ± 51^0,1,2,3^	134 ± 45^0,1,2^
MVIC-R (N)	UGD	1713 ± 507	1235 ± 496^∗∗∗5,7,10^	1279 ± 371^5,7,10^	1334 ± 427^7,10,^	1331 ± 404^5,7,10^	1483 ± 480^0,1,3^	1524 ± 494^0,1,2,3^	1566 ± 466^0,1,2,3^
	GRD	2029 ± 749	1574 ± 595^∗∗∗2,3,5,7,10^	1737 ± 712^7^	1812 ± 685^0^	1830 ± 700^0^	1847 ± 641^0,7^	1931 ± 656^0,1,7^	1885 ± 592^0^
MVIC-L (N)	UGD	1596 ± 539	1168 ± 417^∗∗∗3,5,7,10^	1234 ± 382^5,7,10,^	1314 ± 399	1356 ± 382^0^	1451 ± 443^0,1^	1528 ± 434^0,1^	1520 ± 507^0,1^
	GRD	1986 ± 780	1565 ± 648^∗∗∗1,2,3,5,7,10^	1690 ± 714^0,7^	1784 ± 748^0^	1799 ± 740^0^	1846 ± 727^0^	1892 ± 624^0,1^	1890 ± 600^0^
CK (U ⋅ L^-1^)	UGD	154 ± 99	201 ± 122^∗∗5,7^	641 ± 403^5,7^	548 ± 383^5,7^	638 ± 507	1132 ± 765^0,1,2,10^	653 ± 671^0,1,2,10^	608 ± 422^5,7^
	GRD	260 ± 168	318 ± 237^2^	874 ± 503^1^	482 ± 291	595 ± 570	710 ± 418	465 ± 233	315 ± 273
VAS (mm)	UGD	4 ± 4	37 ± 21^∗∗∗2,10^	51 ± 15^7,10^	69 ± 17^0,5,7,10^	55 ± 17^5,7,10^	26 ± 19^2,3,10^	11 ± 15^1,2,3^	2 ± 2^0,1,2,3,5^
	GRD	5 ± 4	40 ± 29^∗∗∗7,10^	53 ± 26^5,7,10^	65 ± 20^3,5,7,10^	45 ± 22^3,5,7,10^	18 ± 13^1,2,3,7,10^	4 ± 4^0,1,2,3,5^	2 ± 2^0,1,2,3,5,7^

*BL, baseline examination 30 min prior to the intervention; D_*0*_, 5′ post intervention; D_*1*_, D_*2*_, D_*3*_,…, D_*10*_, day 1, day 2, day 3,…, day 10 post intervention; CMJ, counter movement jump; DJ, drop jump; VAS, visual analog scale related to muscle soreness; MVIC, maximal voluntary isometric contraction of the right (R) or left (L) leg; ^∗^,^∗∗^,^∗∗∗^, P < 0.05, P <0.01, P < 0.001 significant difference between baseline (BL) and D_*0*_ (5-min post intervention). ^*0,1,2,…,10*^, significantly to the respective day (0, 1, 2,…,10) post intervention*.

For the DJ jump height, ground contact time and jump coefficient there was a main effect of time (all *P* < 0.001, $\eta_{p}^{2}$ = 0.28 to 0.88, power = 1.0). Both for jump height and ground contact time, no main effect for group was found. Lowest performance in the DJ was detected on day 2 (height and coefficient) and 3 (ground contact time) post intervention. For the jump coefficient a trend toward a group effect (*P* = 0.06) with a lower reduction in GRD compared with UGD (-12.2 ± 10.6% vs. -21.4 ± 10.6%) but no interaction between time x group (*P* = 0.18) was found.

**Figure 3:**
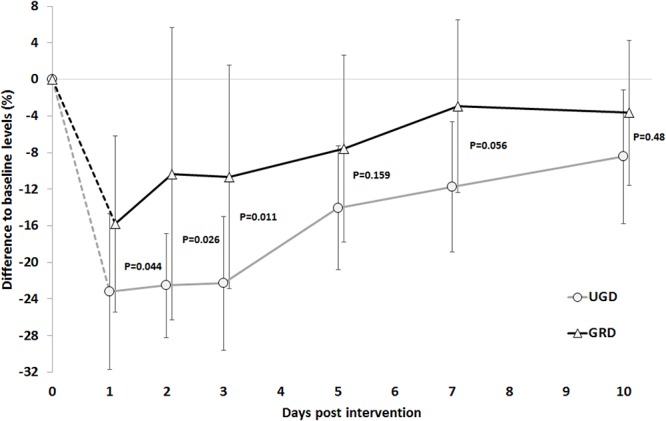
Time course of the % reductions with respect to baseline levels in the isometric maximal strength for the dominant leg across the 10 days post intervention period. UGD, sham-grounded sleeping group; GRD, grounded sleeping group (mean ± SD).

For the MVIC in the dominant leg, a main effect of time (*P* < 0.001, $\eta_{p}^{2}$ = 0.49, power = 1.0) and group (*P* < 0.03, $\eta_{p}^{2}$ = 0.22, power = 0.61) with a less pronounced reduction in performance in GRD compared with UGD (-9.5 ± 16.8% vs. -17.3 ± 38.3%) was found. Day-to-day analysis revealed group × time interactions within the first 3 days with a more pronounced recovery in GRD compared with UGD (*P* < 0.05) (Figure 3). For the non-dominant leg, only a main effect of time was found (*P* < 0.001, $\eta_{p}^{2}$ = 0.49, power = 1.0) with no group differences. Lowest strength performance for both legs was found immediately after the intervention (day 0).

**Figure 4:**
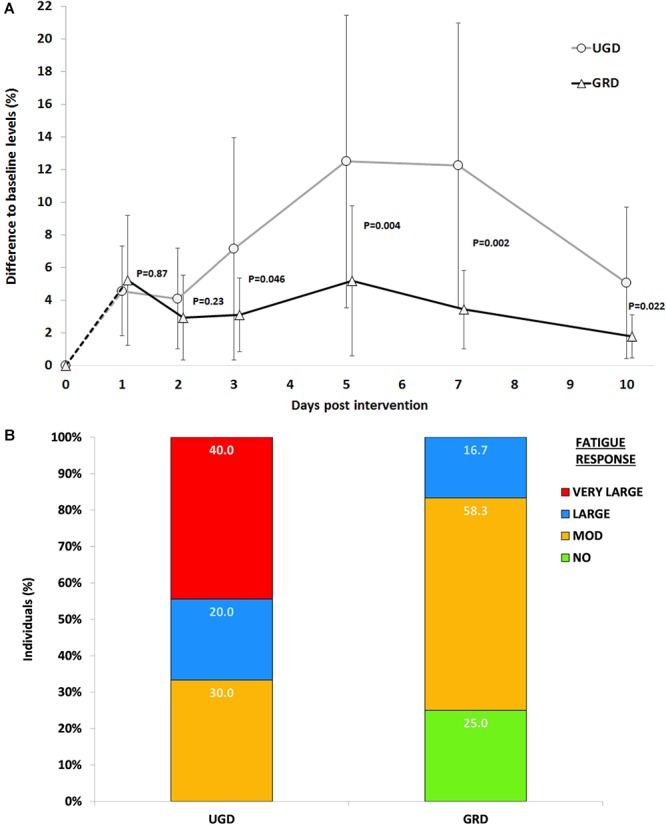
**(A)** Time course of the % increases with respect to baseline levels for CK-blood levels across the 10 days post intervention period. UGD, sham-grounded sleeping group; GRD, grounded sleeping group (mean ± SD). **(B)** Individual response analysis for increases in CK levels with respect to GRD (grounded sleeping) and UGD (sham-grounded sleeping). Percent differences are categorized as non-response: <3%, moderate response: 3–10%, large response: 10–20%, and very large response >20%.

With respect to CK levels main effects of time (*P* < 0.001, $\eta_{p}^{2}$ = 0.30, power = 0.98) and group (*P* = 0.007, $\eta_{p}^{2}$ = 0.31, power = 0.81) and an interaction effect time × group (*P* = 0.001, $\eta_{p}^{2}$ = 0.26, power = 0.95) were found, demonstrating a lower increase GRD compared with UGD (310 ± 120% vs. 760 ± 380%) paralleled with a more pronounced increase in UGD at days 3, 5, and 7. Highest CK levels were measured on day 5 post intervention (Figure 4A). Individual response analysis revealed that within the GRD group none of the participants demonstrated a large increase in CK levels (i.e., change of >20% from baseline), while in UGD this was the case in 40% of the participants (*n* = 4). Furthermore in GRD even 25% (*n* = 3) demonstrated no increase (e.g., <3%) in CK levels while this was not the case in any participant of UGD (Figure 4B).

Regarding VAS only a significant effect of time (*P* < 0.001, $\eta_{p}^{2}$ = 0.55, power = 1.0) with no main effect of group (*P* = 0.13) or interaction effect (*P* = 0.46) was found. VAS was highest at day 2.

**Figure 5:**
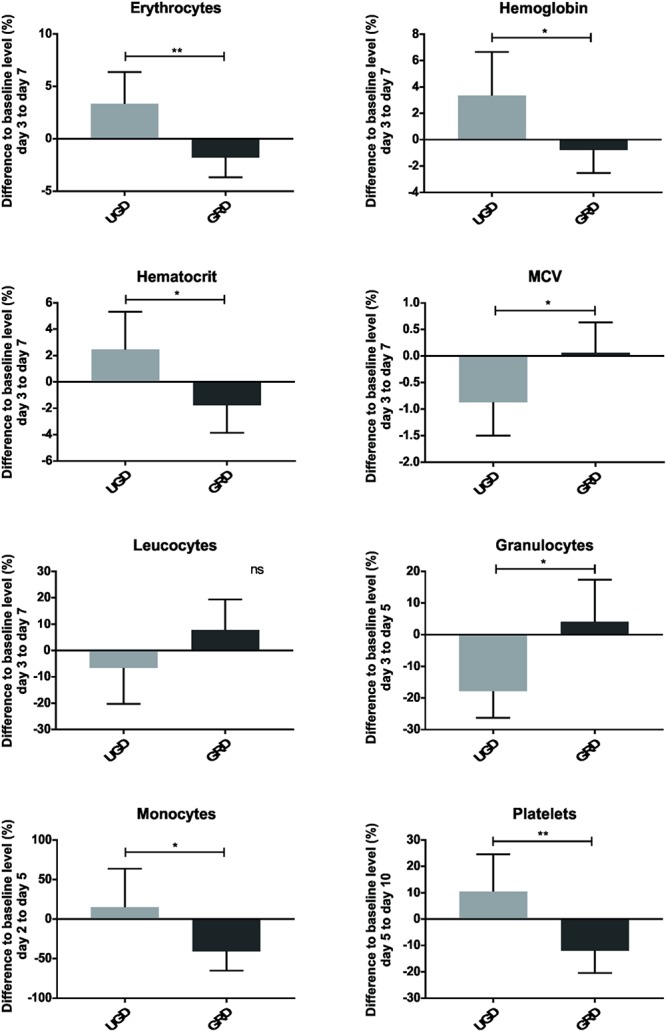
Parameters of the differential blood count observed over a defined time, including erythrocytes, hemoglobin, hematocrit, MCV, leucocytes, granulocytes, monocytes and platelets; represented as mean with standard deviation of the defined days of each group; ^ns^*P* > 0.05, ^∗^*P* ≤ 0.05, ^∗∗^*P* ≤ 0.01, ^∗∗∗^*P* ≤ 0.001.

The effects of grounded sleeping on the post-exercise recovery process of four participants were further analyzed at the cellular and molecular levels. With regard to the differential blood count (Figure 5, Supplementary Figure S1, and Supplementary Tables S1, S2), the most obvious divergence between both groups was observed for erythrocyte counts and for hemoglobin and hematocrit values, which significantly increased from recovery days 2–7 in the UGD group (*P* = 0.007, *P* = 0.029, and *P* = 0.017, respectively). These alterations were accompanied by a decrease in the average volume of red blood cells (MCV, *P* = 0.024). In contrast, these parameters remained at baseline level in the GRD group. No difference was found between the two groups regarding the average mass of hemoglobin per red blood cell (MCH), indicating that the ratio of hemoglobin to erythrocytes was not altered during the recovery process following intense exercise activity. A slight initial boost in the number of leucocytes (10^3^ cells per μl) was observed for the GRD group between days 3 and 7 of the recovery phase (*P* = 0.079). Between recovery days 3 and 5, a similar trend was observed for granulocytes (*P* = 0.038), which represent the vast majority of white blood cells (Lacelle and Cameron, 2015). Interestingly, a significant decrease of blood-derived monocytes was detected in the recovery phase between days 2 and 7 in GRD group (*P* = 0.039). In both UGD and GRD groups, the amount of platelets (10^3^/mm^3^) decreased in the recovery phase following intensive eccentric muscle loading. However, the amount of platelets in the UGD group returned to baseline levels earlier (between days 5 and 10, *P* = 0.01) than in the GRD group, suggesting that a prolonged recruitment of platelets to injured muscle tissue may have occurred. No significant alterations between both groups were observed for the mean corpuscular hemoglobin concentration (MCHC) and the overall lymphocyte count.

The C-reactive protein (CRP) value was monitored throughout the study and used as an indicator of unrelated inflammatory conditions (e.g., viral or bacterial infections), which could affect the evaluated blood parameters. The CRP values remained unaltered in all four subjects (Supplementary Tables S3, S4).

**Figure 6:**
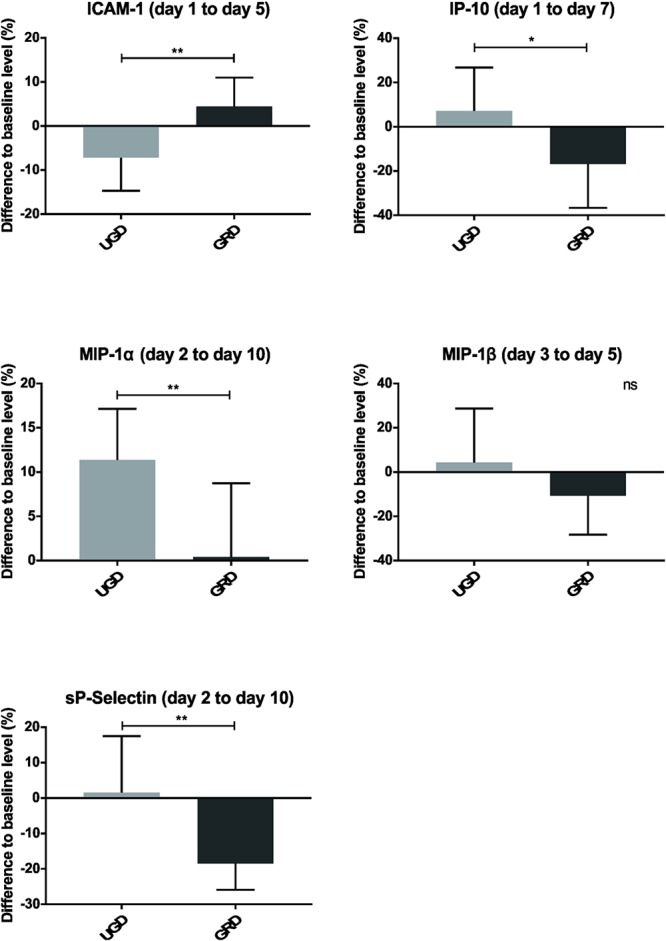
Inflammation markers observed over a defined time, including sICAM-1, IP-10, MIP-1α, MIP-1β, and sP-selectin; represented as mean with standard deviation of the defined days of each group; ^ns^*P* > 0.05, ^∗^*P* ≤ 0.05, ^∗∗^*P* ≤ 0.01, ^∗∗∗^*P* ≤ 0.001.

At the molecular level, we found alterations in serological markers of inflammation, particularly for soluble cell adhesion molecules (sCAM) and chemokines (Figure 6, Supplementary Figure S2, and Supplementary Tables S3, S4). The intercellular adhesion molecule 1 (ICAM-1), which is expressed on epithelial cells and involved in cell adhesion and co-stimulation of macrophages, monocytes and granulocytes, either remained at baseline level or was higher in the GRD compared to the UGD group. From days 1 to 5, a clear decrease of the sICAM-1 levels (approximately 10%) was observed in the UGD group (*P* = 0.005). Furthermore, the cell adhesion molecule, sP-selectin, was downregulated in the GRD compared to the UGD and remained below baseline (approximately 20% reduction; *P* = 0.003) during the whole study period. In general, inflammation-associated chemokines, such as the interferon gamma-induced protein 10 (IP-10) and the macrophage inflammatory proteins (MIP-1α and MIP-1β), showed lower values (*P* = 0.014, *P* = 0.004, and *P* = 0.359, respectively) in the GRD group. No significant alterations were observed for the monocyte chemoattractant protein 1 (MCP-1), which is involved in the recruitment of monocytes, and the sCAM sE-selectin. It was not possible to detect chemokines/cytokines GM-CSF, IFNα, IFNγ, IL-1α, IL-1β, IL-4, IL-6, IL-8, IL-10, IL-12p70, IL-13, IL-17A/CTLA-8, and TNFα in the sera of the healthy athletes within the assay’s limit of detection.

## Discussion

The main findings of the current study were sixfold: (1) the downhill treadmill running intervention led to distinct changes in measured parameters related to fatigue and were already detectable 5 min post intervention; (2) grounded sleeping led in various variables to less pronounced decrease in performance compared with sham-grounded sleeping; (3) grounded sleeping led to a much lower increase of CK compared with UGD during the whole recovery; (4) 10 days post intervention in none of the measured parameters full recovery has been stated; (5) highest CK levels were found on day 5, while for jump and strength performance the highest decrease was the case on day 1 and for VAS the highest increase happened on day 2 post intervention, and (6) detailed blood samples demonstrated that grounded sleeping modulates the recovery process by (a) keeping a constant hemoconcentration, as represented by the number of erythrocytes, and the hemoglobin/hematocrit values, and (b) by the reduction of muscle damage-associated inflammation markers such as IP-10, MIP-1α, and sP-Selectin.

Repetitive eccentric muscle activations like downhill running have repeatedly been shown to be responsible for DOMS. DOMS is associated with release of muscle proteins like CK into the blood stream, as well as with prolonged decreases in muscle performance capacity (Hoppeler, 2016). During eccentric exercises, the activated muscles are stretched which can create mechanical damage to sarcomeres, inflammation, disruption of the sarcolemma, as well as damage by reactive oxygen species (Lovering and Brooks, 2014). The applied 20-min downhill running protocol within the current study led to distinct decreases in performance and increases in measures of muscle damage and VAS, within the majority of analyzed parameters. No measured parameter returned to baseline levels after 10 days of recovery. Therefore, the applied downhill running intervention can be seen as a well-chosen method to provoke muscle damage resulting in distinct decrements in performance. This protocol can, therefore, be seen as suitable for analyzing the effectivity of different types of recovery interventions. In this specific case to analyze if grounded sleeping is able to enhance the ability to recover more quickly, or to dampen the decrements in performance when compared with an ungrounded situation. It is worth noting that Cheung et al. (2003) and Seidel et al. (2012) concluded that, in fact, no treatment strategy consistently supported or enhanced muscle recovery, which indicates that there is actually a lack of compelling evidence-based and practical strategies to help prevent and/or alleviate DOMS. Furthermore, the evidence of treatment strategies to accelerate recovery after EIMD is still inconsistent (Connolly et al., 2003).

However, the current study revealed that GRD showed lower decrements in performance with respect to CMJ, maximal leg strength (MVIC), a trend for DJ coefficient and less pronounced in increase in CK when compared with UGD. The less pronounced decrease in measures of performance (strength, jump performance) and less increase in CK levels within the GRD group might be attributed toward potentially reduced blood viscosity (Chevalier et al., 2013; Brown and Chevalier, 2015), enhanced blood flow velocity, improved sleep quality (Ghaly and Teplitz, 2004) and decreased muscle damage (Brown et al., 2010; Brown et al., 2015) as it is clearly demonstrated by the blood analyses of the present study. Referring to Chevalier et al. (2006); Oschman (2007), and Oschman et al. (2015) the main hypothesis about earthing is based on the connection to the surface of the Earth, which is satiated with free electrons. This indirect or direct contact with the Earth enables “mobile” electrons to migrate into the body. Consequently, Oschman (2007) suggests that the earthing based mobile electrons could also prevent or diminish inflammation, which could also be the reason for the unaffected hemoconcentration as well as the dampened CK response.

The observed increase in the number of erythrocytes and in the hemoglobin and hematocrit values in the course of intensive eccentric muscle loading in the UGD group could be due to volumetric variation. Post-exercise hemoconcentration is typically resulting from water loss (Bloomer and Goldfarb, 2004; Del Coso et al., 2008). Hemoconcentration is also a possible explanation for the increased CK levels in the UGD group (Bassini-Cameron et al., 2007). Our preliminary results suggest that grounded sleeping might prevent the phenomenon of hemoconcentration via the reduction of blood viscosity and improved blood circulation (Chevalier et al., 2013). No alteration in the lymphocyte counts in the blood between both groups was expected since lymphocytes are mostly associated with adaptive immune responses to foreign antigens originating from bacteria, virus, and parasites (Paul and Seder, 1994).

Upon muscle injury, damaged myofibres and other muscle cells at lesion sites undergo necrosis, which in turn are removed by various infiltrating immune cells, mostly mast cells and neutrophils (Bentzinger et al., 2013). These early stages of muscle regeneration are further characterized by activation of the complement system and release of pro-inflammatory cytokines and chemokines such as, TNF-α, IFN-γ, IP-10, and sICAM-1 (Chazaud et al., 2003; Cheng et al., 2008). In response to INF-γ and TNF-α, several cell types secrete interferon gamma-induced protein 10 (IP-10) that can be measured in the blood. The significant decrease in IP-10 observed in the GRD group strongly suggests that grounded sleeping downregulates INF-γ-induced inflammatory responses as well as IP-10-associated (NK) cell-mediated cytolysis. Furthermore, a decrease in the levels of the anti-angiogenic IP-10 might favor angiogenesis and the subsequent influx of blood to the regenerating muscle (Gotsch et al., 2007).

Following these early events in muscle regeneration, muscle stem cells are activated and other immune cells, especially macrophages and T cells, are recruited to the injured muscle tissue. Accordingly, a significant increase in the number of macrophages was observed on day two post-injury (Yang and Hu, 2018). These observations are in line with our findings showing that both chemokines MIP-1α and MIP-1β, which are produced by macrophages (Petray et al., 2002), are increased in the UGD group starting at day 2 post-injury. In contrast, our pilot study showed a decrease in the levels of these chemokines in the GRD group, suggesting a lower attraction of macrophages and thus local inflammation.

Our assumption that grounded sleeping dampens inflammation is further supported by a general decrease of inflammation markers observed in the GRD group. Of note, the sCAM sP-selectin (Schrijver et al., 2017) remained below the baseline level in the GRD group throughout the observation period, suggesting that grounded sleeping has a beneficial and long-lasting effect on this inflammation marker. sP-selectin is found on activated endothelial cells and platelets and is a crucial factor for leukocyte recruitment to lesion sites (Cleator et al., 2006). In the GRD group, a downregulation of activation of sP-selectin on endothelial cells and platelets could result in diminished leukocyte recruitment at sites of damaged muscle tissue.

Our findings that another sCAM, sICAM-1, was found to decline in the UGD compared to the GRD group was not in line with other studies showing high plasma sICAM-1 levels after strenuous exercise. However, sICAM-1 levels seem to be altered depending on the type of exercise as no alterations were observed after bicycle ergometer exercise (Akimoto et al., 2002).

## Conclusion

Taken together, grounded sleeping was shown to result in faster recovery and/or less pronounced markers of muscle damage and inflammation. Our preliminary results with respect to the detailed blood analysis strongly support the view that grounded sleeping modulates key events in the early stages of muscle regeneration at both cellular and molecular levels. Based on the investigated immunological parameters, the modulatory effects of grounded sleeping seem to dampen inflammatory responses triggered by EIMD. GRD might be seen as a simple methodology to enhance acute and long-term recovery after intensive exercises within the training process or following intensive competitions. Based on the results of the pilot study, more research is necessary to clearly point out the mechanisms behind possible effects of grounded sleeping on the cellular and molecular levels. Additionally, the magnitude of being grounded, that is how many “mobile” electrons migrate into the body while GRD vs. UGD sleeping, needs to be investigated further. Finally, whether or not grounded sleeping affects sleep patterns (e.g., sleep quality) also needs to be addressed in future studies. An improvement in the sleeping quality by grounded sleeping might result in alterations in performance and changes in stress markers in athletes.

## Author Contributions

EM, TS, PP, LA, and FF-B conception and designed the experiments. PP and LA performed the experiments. PP, TS, and LA analyzed data. TS, PP, EM, and LA prepared the manuscript. All authors read and approved the final manuscript.

## Conflict of Interest Statement

The authors declare that the research was conducted in the absence of any commercial or financial relationships that could be construed as a potential conflict of interest.
