# Supplementary material for: Effectiveness of Grounded Sleeping on Recovery After Intensive Eccentric Muscle Loading
Source: Front Physiol. 2019 Jan 28;10:35. doi: 10.3389/fphys.2019.00035 (PMC6360250; doi:10.3389/fphys.2019.00035)
Supplement: Supplementary file 1 [file Data_Sheet_1.docx]

**Supplementary Figure S1:** Differential blood count, including erythrocytes, hemoglobin, hematocrit, MCV, MCH, MCHC, leucocytes, monocytes, granulocytes, platelets and lymphocytes; represented as mean with standard deviation.


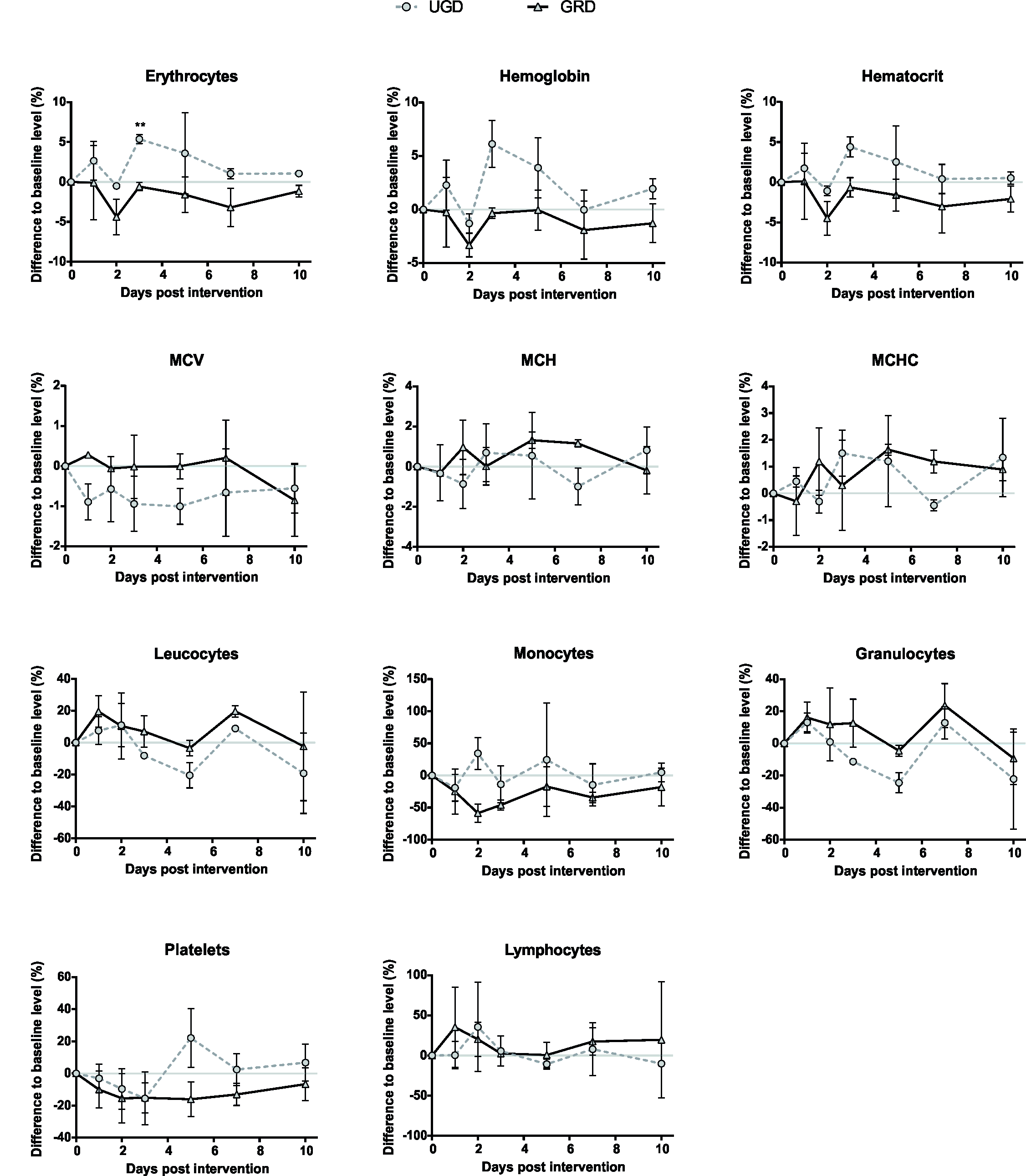


**Supplementary Figure S2:** Analyzed inflammation markers, including sICAM-1, IP-10, MIP-1α, MIP-1β, sE-selectin, sP-selectin and MCP-1; represented as mean with standard deviation.


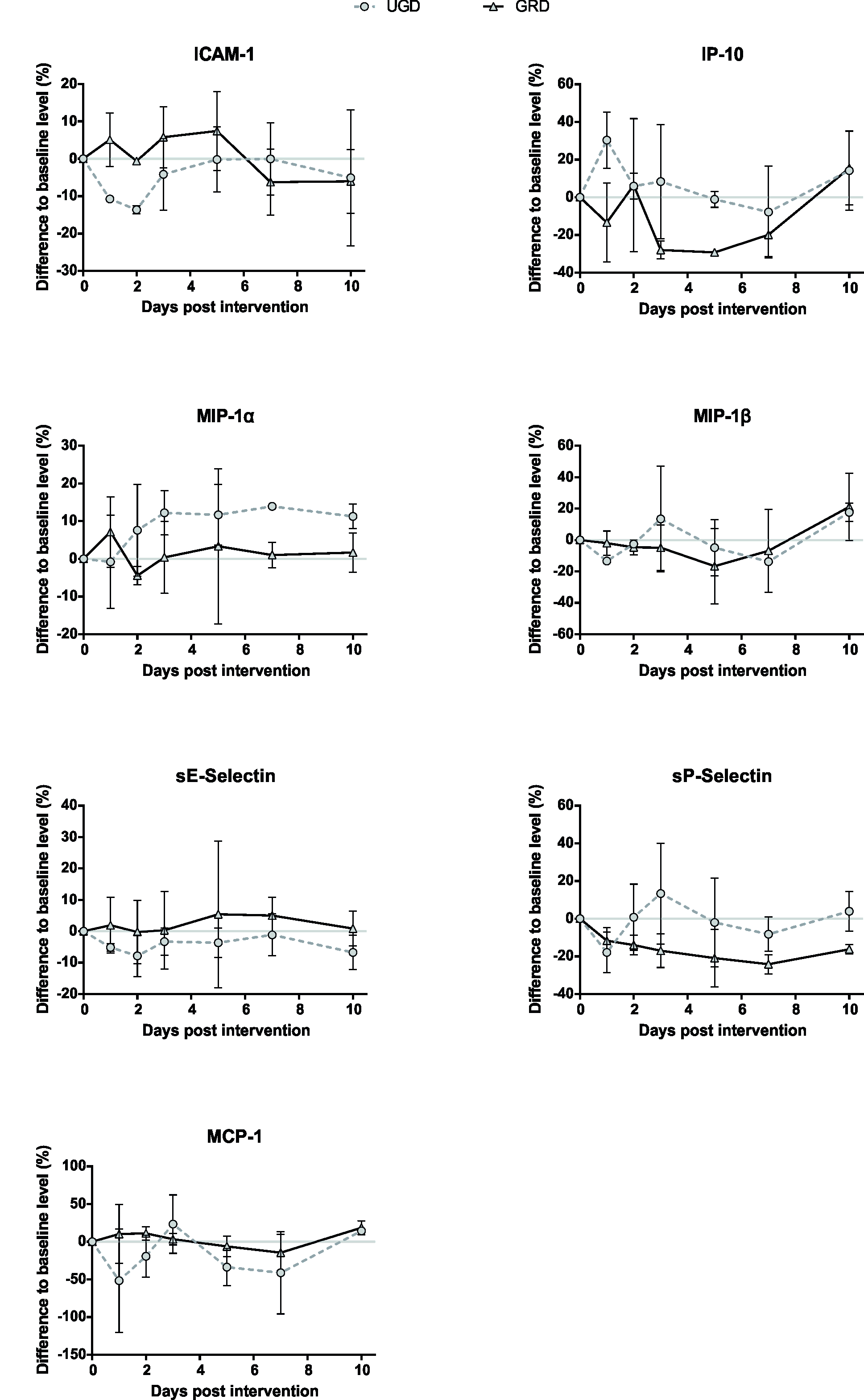


**Table S1:** Differential blood count monitored over the 10 days recovery period in the two participants of the grounded sleeping (GRD) and sham-grounded sleeping (UGD) group. The data represents the individual data of each of the four participants.

|  | **Group** | **BL (-30’)** | **D1 (24 h)** | **D2 (48 h)** | **D3 (72 h)** | **D5** | **D7** | **D10** |  |
| --- | --- | --- | --- | --- | --- | --- | --- | --- | --- |
| Erythrocytes (Mio/µl) | UGD | 5.38 | 5.43 | 5.36 | 5.69 | 5.38 | 5.46 | 5.44 |  |
|  |  | 5.01 | 5.23 | 4.98 | 5.26 | 5.37 | 5.04 | 5.06 |  |
|  | GRD | 4.70 | 4.85 | 4.42 | 4.69 | 4.55 | 4.63 | 4.67 |  |
|  |  | 5.33 | 5.15 | 5.18 | 5.28 | 5.33 | 5.07 | 5.24 |  |
| Hemoglobin (g/dl) | UGD | 15.60 | 15.70 | 15.30 | 16.80 | 15.90 | 15.80 | 15.80 |  |
|  |  | 15.30 | 15.90 | 15.20 | 16.00 | 16.20 | 15.10 | 15.70 |  |
|  | GRD | 14.60 | 14.90 | 14.00 | 14.50 | 14.40 | 14.60 | 14.60 |  |
|  |  | 15.60 | 15.20 | 15.20 | 15.60 | 15.80 | 15.00 | 15.20 |  |
| Hematocrit (%) | UGD | 47.00 | 47.20 | 46.30 | 49.50 | 46.70 | 47.80 | 47.50 |  |
|  |  | 45.60 | 47.00 | 45.30 | 47.20 | 48.20 | 45.20 | 45.60 |  |
|  | GRD | 43.40 | 44.90 | 40.80 | 43.50 | 42.10 | 43.10 | 43.00 |  |
|  |  | 46.60 | 45.10 | 45.20 | 45.90 | 46.50 | 44.10 | 45.10 |  |
| MCV (fl) | UGD | 87.40 | 86.90 | 86.40 | 87.00 | 86.80 | 87.50 | 87.30 |  |
|  |  | 91.00 | 89.90 | 91.00 | 89.70 | 89.80 | 89.70 | 90.10 |  |
|  | GRD | 92.30 | 92.60 | 92.30 | 92.80 | 92.50 | 93.10 | 92.10 |  |
|  |  | 87.40 | 87.60 | 87.30 | 86.90 | 87.20 | 87.00 | 86.10 |  |
| MCH (pg) | UGD | 29.00 | 28.90 | 28.50 | 29.50 | 29.60 | 28.90 | 29.00 |  |
|  |  | 30.50 | 30.40 | 30.50 | 30.40 | 30.20 | 30.00 | 31.00 |  |
|  | GRD | 31.10 | 30.70 | 31.70 | 30.90 | 31.60 | 31.50 | 31.30 |  |
|  |  | 29.30 | 29.50 | 29.30 | 29.50 | 29.60 | 29.60 | 29.00 |  |
| MCHC (%) | UGD | 33.20 | 33.30 | 33.00 | 33.90 | 34.00 | 33.10 | 33.30 |  |
|  |  | 33.60 | 33.80 | 33.60 | 33.90 | 33.60 | 33.40 | 34.40 |  |
|  | GRD | 33.60 | 33.20 | 34.30 | 33.30 | 34.20 | 33.90 | 34.00 |  |
|  |  | 33.50 | 33.70 | 33.60 | 34.00 | 34.00 | 34.00 | 33.70 |  |
| Leucocytes (103/µl) | UGD | 7.30 | 8.30 | 8.80 | 6.80 | 5.40 | 8.00 | 4.60 |  |
|  |  | 7.40 | 7.50 | 7.50 | 6.70 | 6.30 | 8.00 | 7.30 |  |
|  | GRD | 7.20 | 8.10 | 6.90 | 7.20 | 6.70 | 8.80 | 5.30 |  |
|  |  | 6.40 | 8.10 | 8.00 | 7.30 | 6.40 | 7.50 | 7.80 |  |
| Granulocytes (103/µl) | UGD | 5.20 | 6.10 | 5.30 | 4.60 | 3.70 | 5.50 | 2.90 |  |
|  |  | 4.50 | 4.90 | 4.50 | 4.00 | 3.60 | 5.40 | 4.50 |  |
|  | GRD | 4.80 | 5.90 | 4.60 | 4.90 | 4.70 | 6.40 | 3.80 |  |
|  |  | 4.30 | 4.70 | 5.50 | 5.30 | 4.00 | 4.90 | 4.40 |  |
| Monocytes (%) | UGD | 7.10 | 4.70 | 8.30 | 4.70 | 4.40 | 4.40 | 6.70 |  |
|  |  | 4.60 | 4.40 | 7.00 | 4.90 | 8.60 | 5.00 | 5.30 |  |
|  | GRD | 7.20 | 3.60 | 3.70 | 3.50 | 4.40 | 4.30 | 4.40 |  |
|  |  | 6.70 | 6.70 | 2.10 | 4.00 | 7.00 | 4.80 | 6.90 |  |
| Platelets (103/mm3) | UGD | 151 | 137 | 123 | 110 | 204 | 144 | 149 |  |
|  |  | 282 | 291 | 280 | 271 | 308 | 309 | 324 |  |
|  | GRD | 330 | 270 | 243 | 258 | 252 | 271 | 284 |  |
|  |  | 329 | 323 | 314 | 301 | 301 | 302 | 331 |  |
| Lymphocytes (103/µl) | UGD | 1.60 | 1.80 | 2.80 | 1.90 | 1.50 | 2.10 | 1.40 |  |
|  |  | 2.60 | 2.30 | 2.50 | 2.40 | 2.20 | 2.20 | 2.40 |  |
|  | GRD | 1.90 | 1.90 | 2.00 | 2.00 | 1.70 | 2.00 | 1.30 |  |
|  |  | 1.70 | 2.90 | 2.30 | 1.70 | 1.90 | 2.20 | 2.90 |  |

BL, baseline examination 30 min prior to the intervention; D1, D2, D3,…, D10, day 1, day 2, day 3,…, day 10 post intervention; MCV, average volume of a red blood cell values; MCH, average mass of hemoglobin per red blood cell; MCHC, mean corpuscular hemoglobin concentration.

**Table S2:** Normalized differential blood count values (mean ± SD).

|  | **Group** | **D1 (24 h)** | **D2 (48 h)** | **D3 (72 h)** | **D5** | **D7** | **D10** |  |
| --- | --- | --- | --- | --- | --- | --- | --- | --- |
| Erythrocytes | UGD | 2.7±2.4 | -0.5±0,2 | 5.4±0.5 | 3.6±5.1 | 1.0±0.6 | 1.1±0.1 | |
|  | GRD | -0.1±4.6 | -4.4±2.2 | -0.6±0.5 | -1.6±2.3 | -3.2±2.4 | -1.2±0.7 | |
| Hemoglobin | UGD | 2.3±2.3 | -1.3±0.9 | 6.1±2.2 | 3.9±2.8 | 0.0±1.8 | 1.9±0.9 | |
|  | GRD | -0.3±3.3 | -3.3±1.1 | -0.3±0.5 | 0.0±1.9 | -1.9±2.7 | -1.3±1.8 | |
| Hematocrit | UGD | 1.7±1.9 | -1.1±0.6 | 4.4±1.3 | 2.5±4.5 | 0.4±1.8 | 0.5±0.8 | |
|  | GRD | 0.1±4.7 | -4.5±2.1 | -0.6±1.2 | -1.6±2.0 | -3.0±3.3 | -2.1±1.6 | |
| MCV | UGD | -0.9±0.5 | -0.6±0.8 | -0.9±0.7 | -1.0±0.4 | -0.7±1.1 | -0.6±0.6 | |
|  | GRD | 0.3±0.1 | -0.1±0.1 | 0.0±0.8 | 0.0±0.3 | 0.2±0.9 | -0.9±0.9 | |
| MCH | UGD | -0.3±0.0 | -0.9±1.2 | 0.7±1.5 | 0.5±2.2 | -1.0±0.9 | 0.8±1.2 | |
|  | GRD | -0.3±1.4 | 1.0±1.4 | 0.0±0.9 | 1.3±0.4 | 1.2±0.2 | -0.2±1.2 | |
| MCHC | UGD | 0.4±0.2 | -0.3±0.4 | 1.5±0.9 | 1.2±1.7 | -0.4±0.2 | 1.3±1.5 | |
|  | GRD | -0.3±1.3 | 1.2±1.3 | 0.3±1.7 | 1.6±0.2 | 1.2±0.4 | 0.9±0.4 | |
| Leucocytes | UGD | 7.5±8.7 | 10.9±13.6 | -8.2±1.8 | -20.4±7.9 | 8.8±1.0 | -19.2±25.2 | |
|  | GRD | 19.5±9.9 | 10.4±20.6 | 7.0±9.9 | -3.5±4.9 | 19.7±3.6 | -2.3±34.1 | |
| Granulocytes | UGD | 13.1±6.0 | 1.0±1.4 | -11.3±0.3 | -24.4±6.3 | 12.9±10.1 | -22.1±31.1 | |
|  | GRD | 16.1±9.6 | 11.9±22.7 | 12.7±15.0 | -4.5±3.5 | 23.6±13.7 | -9.3±16.4 | |
| Monocytes | UGD | -19.1±20.8 | 34.5±24.9 | -13.6±28.5 | 24.5±88.4 | -14.7±33.0 | 4.8±14.7 | |
|  | GRD | -25.0±35.4 | -58.6±14.2 | -45.8±7.8 | -17.2±30.7 | -34.3±8.4 | -18.0±29.6 | |
| Platelets | UGD | -3.0±8.8 | -9.6±12.6 | -15.5±16.4 | 22.2±18.3 | 2.5±10.0 | 6.8±11.5 | |
|  | GRD | -10.0±11.6 | -15.5±15.4 | -15.2±9.4 | -16.1±10.7 | -13.0±6.8 | -6.7±10.3 | |
| Lymphocytes | UGD | 0.5±17 | 35.6±55.8 | 5.5±18.7 | -10.8±6.5 | 7.9±33.0 | -10.1±3.4 | |
|  | GRD | 35.3±49.9 | 20.3±21.2 | 2.6±3.7 | 0.6±15.8 | 17.3+17.1 | 19.5±72.2 | |

D1, D2, D3,…, D10, day 1, day 2, day 3,…, day 10 post intervention; MCV, average volume of a red blood cell values; MCH, average mass of hemoglobin per red blood cell; MCHC, mean corpuscular hemoglobin concentration.

**Table S3:** Serological inflammation markers (pg/ml) over the 10 days recovery period in the two participants of the grounded sleeping (GRD) and sham-grounded sleeping (UGD) group. The data represents the individual data of each of the four participants.

|  | **Group** | **BL (-30’)** | **D1 (24 h)** | **D2 (48 h)** | **D3 (72 h)** | **D5** | **D7** | **D10** |
| --- | --- | --- | --- | --- | --- | --- | --- | --- |
| ICAM-1 | UGD | 61039.40 | 54211.61 | 53178.89 | 54375.14 | 57147.61 | 56858.51 | 50104.49 |
|  |  | 37745.74 | 33862.06 | 32327.60 | 38723.92 | 39999.09 | 40324.28 | 40677.26 |
|  | GRD | 3905873.00 | 3905873.00 | 3905873.00 | 3905873.00 | 3905873.00 | 3905873.00 | 3905873.00 |
|  |  | 51346.14 | 56568.31 | 50707.06 | 57289.92 | 58996.55 | 44932.25 | 45165.30 |
| sP-selectin | UGD | 70059.82 | 52226.65 | 61887.08 | 66174.46 | 57069.72 | 59779.61 | 67565.52 |
|  |  | 67234.63 | 60357.96 | 76127.97 | 88943.88 | 77092.85 | 66027.15 | 74909.56 |
|  | GRD | 103421.00 | 86217.95 | 85130.15 | 79356.68 | 70648.19 | 82222.02 | 84785.46 |
|  |  | 91078.20 | 84981.31 | 81802.15 | 81354.46 | 81906.99 | 65843.55 | 77897.27 |
| sE-selectin | UGD | 10767.71 | 10313.16 | 10429.78 | 10084.03 | 10020.29 | 10131.97 | 9620.95 |
|  |  | 17454.02 | 16403.27 | 15272.14 | 17422.56 | 17391.55 | 18086.90 | 16959.53 |
|  | GRD | 16426.81 | 15717.16 | 15210.96 | 15048.22 | 14608.76 | 16574.49 | 15924.86 |
|  |  | 21245.10 | 22982.16 | 22711.90 | 23173.49 | 25909.05 | 23181.38 | 22259.88 |
| IP-10 | UGD | 317.84 | 447.92 | 352.13 | 276.30 | 324.09 | 347.77 | 410.29 |
|  |  | 314.78 | 377.10 | 318.11 | 408.72 | 301.82 | 235.93 | 312.42 |
|  | GRD | 299.22 | 303.54 | 243.53 | 205.29 | 214.26 | 264.38 | 386.80 |
|  |  | 114.84 | 82.49 | 150.96 | 86.60 | 80.44 | 82.49 | 116.68 |
| MIP-1α | UGD | 16.84 | 15.24 | 16.67 | 18.20 | 17.85 | 19.20 | 18.35 |
|  |  | 20.29 | 21.90 | 23.58 | 23.62 | 23.82 | 23.11 | 23.04 |
|  | GRD | 9.47 | 9.52 | 9.21 | 8.87 | 8.41 | 9.79 | 9.28 |
|  |  | 8.94 | 10.17 | 8.39 | 9.58 | 10.54 | 8.82 | 9.42 |
| MIP-1β | UGD | 91.79 | 79.35 | 89.65 | 82.33 | 75.76 | 77.84 | 111.82 |
|  |  | 67.76 | 58.81 | 65.83 | 92.96 | 73.04 | 59.45 | 76.86 |
|  | GRD | 82.01 | 84.88 | 75.43 | 69.62 | 54.42 | 91.70 | 111.80 |
|  |  | 61.58 | 57.02 | 60.76 | 64.95 | 61.83 | 45.84 | 65.28 |
| MCP-1 | UGD | 94.47 | 91.49 | 95.08 | 90.78 | 79.28 | 92.06 | 108.06 |
|  |  | 6.59 | 0.00 | 4.02 | 9.94 | 3.22 | 1.34 | 25.83 |
|  | GRD | 79.44 | 109.61 | 83.30 | 86.47 | 66.80 | 81.65 | 99.38 |
|  |  | 174.38 | 143.79 | 204.80 | 171.18 | 181.01 | 119.04 | 195.33 |
| CRP | UGD | 0.38 | 0.52 | 0.24 | 0.19 | 0.18 | 0.20 | 0.17 |
|  |  | 0.19 | 0.19 | 0.18 | 0.19 | 0.20 | 0.19 | 0.18 |
|  | GRD | 0.18 | 0.19 | 0.18 | 0.19 | 0.17 | 0.18 | 0.18 |
|  |  | 0.20 | 0.19 | 0.19 | 0.19 | 0.20 | 0.18 | 0.20 |

D1, D2, D3,…, D10, day 1, day 2, day 3,…, day 10 post intervention; intercellular adhesion molecule 1, ICAM-1; serum platelets/endothelial cells selectin, sP/sE-selectin; interferon gamma-induced protein 10, IP-10; macrophage inflammatory proteins, MIP; monocyte chemoattractant protein 1, MCP-1; C-reactive protein, CRP.

**Table S4:** Normalized serological inflammation markers (mean ± SD).

|  | **Group** | **D1 (24 h)** | **D2 (48 h)** | **D3 (72 h)** | **D5** | **D7** | **D10** |
| --- | --- | --- | --- | --- | --- | --- | --- |
| ICAM-1 | UGD | -10.74±0.63 | -13.62±1.04 | -4.16±9.55 | -0.20±8.73 | -0.01±9.67 | -5.07±18.16 |
|  | GRD | 5.09±7.19 | -0.62±0.88 | 5.79±8.19 | 7.45±10.54 | -6.25±8.83 | -6.02±8.51 |
| sP-selectin | UGD | -17.84±10.77 | 0.78±17.60 | 13.37±26.75 | -1.94±23.48 | -8.23±9.11 | 3.93±10.59 |
|  | GRD | -11.66±7.03 | -13.94±5.30 | -16.97±8.90 | -20.88±15.29 | -24.10±5.10 | -16.25±2.51 |
| sE-selectin | UGD | -5.12±1.27 | -7.82±6.62 | -3.26±4.36 | -3.65±4.66 | -1.14±6.74 | -6.74±5.53 |
|  | GRD | 1.93±8.84 | -0.25±10.12 | 0.34±12.35 | 5.44±23.35 | 5.01±5.81 | 0.86±5.54 |
| IP-10 | UGD | 30.36±14.94 | 5.92±6.88 | 8.39±30.34 | -1.08±4.30 | -7.82±24.37 | 14.17±21.10 |
|  | GRD | -13.36±20.94 | 6.42±35.40 | -27.99±4.81 | -29.17±1.10 | -19.91±11.69 | 15.44±19.56 |
| MIP-1α | UGD | -0.78±12.33 | 7.60±12.18 | 12.24±5.89 | 11.70±8.06 | 13.96±0.08 | 11.26±3.24 |
|  | GRD | 7.14±9.36 | -4.45±2.41 | 0.41±9.54 | 3.35±20.57 | 1.02±3.34 | 1.68±5.22 |
| MIP-1β | UGD | -13.38±0.24 | -2.59±0.37 | 13.44±33.58 | -4.84±17.86 | -13.73±2.07 | 17.63±5.93 |
|  | GRD | -1.95±7.71 | -4.68±4.73 | -4.82±14.55 | -16.62±24.08 | -6.87±26.43 | 21.17±21.44 |
| MCP-1 | UGD | -51.58±68.48 | -19.18±28.03 | 23.46±38.71 | -33.61±24.79 | -41.11±54.53 | 153.17±196.27 |
|  | GRD | 10.22±39.26 | 11.15±8.90 | 3.51±7.56 | -6.05±13.94 | -14.48±24.41 | 18.56±9.25 |

D1, D2, D3,…, D10, day 1, day 2, day 3,…, day 10 post intervention; intercellular adhesion molecule 1, ICAM-1; serum platelets/endothelial cells selectin, sP/sE-selectin; interferon gamma-induced protein 10, IP-10; macrophage inflammatory proteins, MIP; monocyte chemoattractant protein 1, MCP-1.
